# Availability and Suitability of Digital Health Tools in Africa for Pandemic Control: Scoping Review and Cluster Analysis

**DOI:** 10.2196/30106

**Published:** 2021-12-23

**Authors:** Bernard C Silenou, John L Z Nyirenda, Ahmed Zaghloul, Berit Lange, Juliane Doerrbecker, Karl Schenkel, Gérard Krause

**Affiliations:** 1 Department of Epidemiology, Helmholtz Centre for Infection Research Braunschweig Germany; 2 PhD Programme Epidemiology, Braunschweig-Hannover Hannover Germany; 3 Department of Infectious Diseases, University Hospital Freiburg, Medical Faculty, University of Freiburg Freiburg Germany; 4 Africa Centres for Disease Control and Prevention Addis Ababa Ethiopia; 5 German Center for Infection Research Braunschweig Germany; 6 World Health Organization Geneva Switzerland

**Keywords:** mobile applications, mHealth, epidemiological surveillance, communicable diseases, outbreak response, health information management, public health, review, transmission network

## Abstract

**Background:**

Gaining oversight into the rapidly growing number of mobile health tools for surveillance or outbreak management in Africa has become a challenge.

**Objective:**

The aim of this study is to map the functional portfolio of mobile health tools used for surveillance or outbreak management of communicable diseases in Africa.

**Methods:**

We conducted a scoping review by combining data from a systematic review of the literature and a telephone survey of experts. We applied the PRISMA (Preferred Reporting Items for Systematic Reviews and Meta-Analyses) guidelines by searching for articles published between January 2010 and December 2020. In addition, we used the respondent-driven sampling method and conducted a telephone survey from October 2019 to February 2020 among representatives from national public health institutes from all African countries. We combined the findings and used a hierarchical clustering method to group the tools based on their functionalities (attributes).

**Results:**

We identified 30 tools from 1914 publications and 45 responses from 52% (28/54) of African countries. Approximately 13% of the tools (4/30; Surveillance Outbreak Response Management and Analysis System, Go.Data, CommCare, and District Health Information Software 2) covered 93% (14/15) of the identified attributes. Of the 30 tools, 17 (59%) tools managed health event data, 20 (67%) managed case-based data, and 28 (97%) offered a dashboard. Clustering identified 2 exceptional attributes for outbreak management, namely *contact follow-up* (offered by 8/30, 27%, of the tools) and *transmission network visualization* (offered by Surveillance Outbreak Response Management and Analysis System and Go.Data).

**Conclusions:**

There is a large range of tools in use; however, most of them do not offer a comprehensive set of attributes, resulting in the need for public health workers having to use multiple tools in parallel. Only 13% (4/30) of the tools cover most of the attributes, including those most relevant for response to the COVID-19 pandemic, such as laboratory interface, contact follow-up, and transmission network visualization.

## Introduction

### Background

In 1998, following the resolution of the 48th World Health Assembly, the African Region of the World Health Organization (WHO) approved Integrated Disease Surveillance and Response (IDSR) for all member states in Africa to adopt as the main strategy for strengthening national disease surveillance systems [1,2]. The IDSR system is a regular and continuous reporting of surveillance data for priority epidemic-prone diseases by the health facilities from the district to national or state levels of the health system using paper case forms [2]. The IDSR makes it possible for countries to be able to identify and contain disease outbreaks. Nonetheless, countries using the IDSR system face delays in transferring data from one level to another, are error-prone with low completeness, and face difficulty in updating data after it has been posted from one level to another [3].

The ubiquitous development of mobile health (mHealth) surveillance tools to alleviate the challenges faced by the paper-based IDSR has resulted in a large variety of tools in the field. Researchers have conducted studies to review the properties of and challenges faced by mHealth intervention programs [4-6]. However, a knowledge gap exists in the extent to which these tools are similar to each other and the relevant minimum set of attributes these tools share. Thus, assessing and mapping tools according to certain requirements and attributes will be of great importance to international organizations, public health stakeholders, and mHealth development teams.

### Objective

The aim of this study is to map mHealth tools used in Africa for surveillance or outbreak management of communicable diseases to identify commonalities among tools, propose recommendations for further development of mHealth tools, and discuss countries’ needs.

## Methods

We established a 2-stage systematic scoping approach by combining data from an mHealth tool user telephone survey and a literature search (peer-reviewed or gray literature). To reduce bias and increase coverage of identified tools, we combined these approaches, as many mHealth tools may not be covered in scientific publications, and those that are covered may not be in use in any of the African countries [7].

### Survey

In the first stage, we created a list of 15 relevant attributes by combining information from the following sources: (1) the Technical Guidelines for Integrated Disease Surveillance and Response in the African Region [8], (2) the WHO Ebola Virus Disease Consolidated Preparedness Checklist [9], and (3) authors’ expert-based knowledge and experiences with communicable disease surveillance. The first 2 sources reflect contributions from >100 public health experts from the WHO, the Centers for Disease Control and Prevention, the United Nations Office for the Coordination of Humanitarian Affairs, and ministries of health in African countries. Figure S2 in Multimedia Appendix 1 shows the flowchart of how we extracted the relevant attributes, whereas Table 1 and Multimedia Appendix 1, Table S2 present the definitions of the attributes included and excluded from the study, respectively. Furthermore, we used the respondent-driven sampling method and conducted a telephone survey on the relevant attributes of mHealth tools used in Africa [10].

To recruit the interviewees, we designed a short email questionnaire and sent it (through the Africa Centers for Disease Control and Prevention official email), in May 2019, to each surveillance focal point or state epidemiologist of all 54 member states of the African Union. The question in the email requested the (1) names of electronic tools used for disease surveillance or outbreak management in their respective countries, (2) contact details of relevant stakeholders who could participate in a telephone survey, and (3) websites of the tools if available. To the countries that did not respond to the initial questionnaire, we sent a reminder email 2 weeks later. Subsequently, we assembled the collective responses and developed a database comprising stakeholders and owners of the tools, whom we then contacted to take part in the telephone survey. We assessed 15 relevant attributes during a telephone survey conducted in English or French from stakeholders who were successfully contacted and willing to participate. Stakeholders who could not be contacted or refused to participate were dropped. Each telephone survey lasted 15 minutes on average. We requested interviewees to provide additional contact information of persons who might be able to provide more information on the respective tool or on other tools for which we had not yet conducted interviews. The respondent-driven sampling and subsequent telephone interviews were conducted by BCS from October 2019 to February 2020.

### Literature Search

In the second stage, we searched for articles published from January 1, 2010, to December 24, 2020, on MEDLINE via the PubMed interface and Google Scholar using the PRISMA (Preferred Reporting Items for Systematic Reviews and Meta-Analyses) guidelines as a search strategy [11]. We included articles on digital apps designed for communicable disease surveillance used in Africa during the period from 2010 to 2020. We excluded articles on digital apps without a mobile component or those used solely for noncommunicable diseases, patient management, or laboratory management.

We developed the search strategy on PubMed by first identifying keywords corresponding to our inclusion criteria and subsequently performing a series of search tests using these terms and Boolean operators. We then identified the search with 100% sensitivity based on a predefined set of 10 articles on mHealth tools known to the authors. The exact query used on December 26, 2020 on PubMed was *((Mobile Applications)* OR *(Digital Applications)* OR *mHealth* OR *eHealth)* AND *((Public Health)* OR *(Communicable Diseases)* OR *Surveillance)* AND *Africa,* while that used on Google Scholar was *Africa* AND *surveillance* AND *(Mobile Applications)* OR *(digital applications)* AND *(Infectious diseases)*.

After the deduplication of the records, BCS and JLZN independently screened the titles and abstracts and included only those articles that mentioned any electronic tool for communicable disease surveillance. We then extracted a set of unique tools from the collection of eligible articles. Subsequently, BCS and JLZN independently selected the tools that met the inclusion criteria using information from the respective publications and websites of these tools.

### Data Extraction

**Figure 1:**
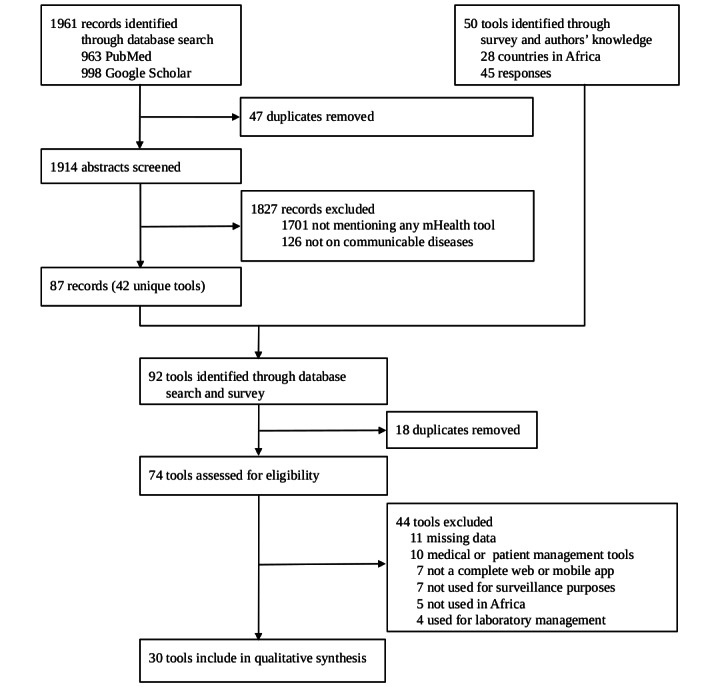
Flowchart to identify electronic tools for communicable disease surveillance used in Africa from January 2010 to December 2020.

The 2 authors then mapped the selected tools based on the list of 15 relevant attributes using the information found in the articles and on the websites of the tools. The literature search and subsequent mapping of the tools spanned from November 2020 to January 2021. Subsequently, we merged the findings from the survey with those from the literature review to construct the data for analysis. The data comprised 30 rows (tools) and 15 columns (attributes; Table 1). The value of each cell in the data was 1 (if the tool had the attribute), 0 (if the tool did not have the attribute), or blank (if missing). Figure 1 shows a flowchart of how we identified the tools included in this study.

**Table 1:** Number and proportion of 15 relevant attributes of 30 electronic tools for communicable diseases surveillance used in Africa from January 2010 to December 2020.

Attribute label and type				Attribute description		Number of tools with available data for attribute (N=30)		Number of tools having attribute, n (%)
**Offline^a^**								
	Nonfunctional		The tool functions without a connection to the internet and synchronizes with the server when connectivity is available		30		29 (97)	
**Dashboard^b^**								
	Nonfunctional		The tool has a dashboard to display epidemiological indicators and charts		29		28 (97)	
**Synchronization^a^**								
	Nonfunctional		The tool can synchronize with the server using 3G, 4G, or WiFi		30		27 (90)	
**Aggregate^b^**								
	Functional		The tool can be used for monthly or weekly aggregate and zero reporting (tally tool)		29		25 (86)	
**Geolocation^b^**								
	Nonfunctional		The tool can document and display the exact geolocation of entities on a map (eg, cases, contacts, and events)		30		24 (80)	
**Open source^a^**								
	Nonfunctional		The source code of the tool is publicly available, and software can be used without license fees		26		22 (85)	
**Case** **-** **based^b, c^**								
	Functional		The tool can be used for case-based reporting (line listing tool)		30		20 (67)	
**Events^b, c^**								
	Functional		The tool can document data on disease events (ie, event-based surveillance)		29		17 (59)	
**Flexible form^a^**								
	Nonfunctional		The tool has a flexible form builder for users to design questions and the type of data to collect		27		13 (48)	
**Attributes that apply to case-based tools only^d^**								
	**Symptom^b^**							
		Functional	The tool documents symptoms for cases		20		20 (100)	
	**Exposure^b^**							
		Functional	The tool documents epidemiological data (eg, exposures to other cases, animal contacts, and travel history) for cases		20		19 (95)	
	**Hospitalization^b^**							
		Functional	The tool documents hospitalization for cases		20		19 (95)	
	**Laboratory^b, c^**							
		Functional	The tool has a laboratory interface to document samples and their test results		19		13 (68)	
	**Contact follow-up^b, c^**							
		Functional	The tool can document and track chains of transmission by linking contacts to cases and document follow-up data (contact tracing)		20		8 (40)	
	**Transmission network^a^**							
		Nonfunctional	The tool has a feature for visualizing and exploring chains of transmission (disease transmission network)		19		2 (11)	

^a^Identified through expert-based knowledge and experiences with communicable diseases surveillance.

^b^Identified from the World Health Organization’s Technical Guidelines for Integrated Disease Surveillance and Response in the African Region [8].

^c^Identified from the World Health Organization’s Ebola Virus Disease Consolidated Preparedness Checklist [9].

^d^N=20.

### Data Analysis

To explore the similarities among the tools in terms of the relevant attributes, we visualized the data using hierarchical clustering [12]. As the data comprised binary variables only, we adopted the Jaccard similarity measure and complete linkage method to construct the dendrogram [13]. We used the constant-height tree cut method with a minimum cluster side of 1 to group the branches of the dendrogram [14]. To visualize the tools and attributes in the same figure, we used a heat map and clustered the data in such a manner that similar tools and attributes were assembled together [15]. We also calculated the proportion of tools that shared each attribute. From the responses obtained from the survey, we derived categories with frequencies for the challenges and needs to sustain mHealth programs in these countries. We used R (R Core Team) and the ComplexHeatmap package in this analysis [15].

## Results

### Tools and Associated Attributes

The telephone survey had 45 full responses from 28 countries. Table S1 in Multimedia Appendix 1 shows the countries that responded to the telephone survey and their corresponding number of responses. The number of respondents per country ranged from 1 to 3 (Table S1 in Multimedia Appendix 1). Of the 28 countries, 26 (93%) countries used specific electronic tools for surveillance, and 2 (7%) countries did not have a specific electronic tool but used telephone calls, email, and WhatsApp to transfer surveillance data from one level to another.

We evaluated 15 relevant attributes from 30 tools identified through a literature search (from 1914 records) and a survey (Figure 1). Surveillance tools most frequently used in >1 country based on the results of the telephone survey were District Health Information Software 2 (DHIS2; used by 23/26, 88% of the countries), Early Warning, Alert, and Response System (6/26, 23% countries), Auto-Visual Acute flaccid paralysis Detection And Reporting (5/26, 19% countries), Epi Info (4/26, 15% countries), Surveillance Outbreak Response Management and Analysis System (SORMAS; 2/26, 8% countries), and electronic IDSR (2/26, 8% countries). Of the 30 tools, 3 (10%) were developed and used only in 1 country each: Notifiable Medical Conditions Surveillance System in South Africa, Meningitis Platform in Morocco, and District Health Information Management System in Ghana. In addition, 10% (3/30) of the tools were designed to manage only 1 disease: Coconut Surveillance for malaria, Meningitis Platform for meningitis, and Auto-Visual Acute flaccid paralysis Detection And Reporting for acute flaccid paralysis. Regarding the broadly implemented DHIS2, the participating countries used it for different purposes, namely aggregate surveillance, case-based surveillance, or as a data warehouse or data storage for vaccination campaigns and infrastructural data. On the basis of available data, the number of tools covering each specific attribute ranged from 2 to 29 (Table 1). Offline mode and dashboard were the attributes covered by 97% (28/30) tools, whereas contact follow-up and transmission network were included in 11% (2/30) of the tools (Table 1). The number of attributes for each of the 30 identified tools ranged from 3 to 14 (Table 2).

**Table 2:** Description of 30 electronic tools for communicable diseases surveillance used in Africa from January 2010 to December 2020.

Name of tool and description^a^		Society affiliation	Attributes^b^ (N=15), n (%)
**DHIS2^c^ [16]**			
	DHIS is an open-source software platform for the reporting, analysis, and dissemination of data for all health programs. DHIS2 is typically used as a national health information system for data management and analysis purposes, for health program monitoring and evaluation, as facility registries and service availability mapping, and for logistics management, and for mobile tracking of pregnant mothers in rural communities.	The Health Information Systems Program at the University of Oslo	14 (93)
**SORMAS^d^ [17-20]**			
	SORMAS is an open-source mobile eHealth system that processes disease control and outbreak management procedures in addition to surveillance and early detection of outbreaks through real-time digital surveillance, including peripheral health care facilities and laboratories. SORMAS adheres to data standards and enhances technical and contextual interoperability with other systems.	Helmholtz Centre for Infection Research, Braunschweig, Germany	14 (93)
**Go.Data [21]**			
	Go.Data is an outbreak investigation tool for field data collection during public health emergencies. The tool includes functionality for case investigation, contact follow-up, and visualization of chains of transmission, including secure data exchange, and is designed for flexibility in the field to adapt to the wide range of outbreak scenarios. The tool is targeted at any outbreak responder.	World Health Organization	14 (93)
**CommCare [22]**			
	CommCare is an open-source mobile platform designed for data collection, client management, decision support, and behavior change communication. It is used by client-facing community health workers during visits as a data collection and educational tool and includes optional audio, image, and video prompts.	Dimagi, Inc Mobile Solutions for International Development	14 (93)
**Epi Info [23]**			
	Epi Info is a public-domain suite of interoperable software tools designed for the global community of public health practitioners and researchers. It provides easy data entry forms and database construction, a customized data entry experience, and data analyses with epidemiological statistics, maps, and graphs for public health professionals who may lack an information technology background.	Centers for Disease Control and Prevention (CDC)	13 (87)
**eIDSR^e^ [24]**			
	eIDSR is an electronic disease surveillance and response system using mobile technology and interactive voice response. eIDSR was developed by leveraging the expertise of DHIS2 to customize a comprehensive eIDSR module on the platform.	The Ministry of Health Rwanda Biomedical Center and USAID’s Rwanda Health Systems Strengthening Project led by Management Sciences for Health	13 (87)
**KoBoToolbox [25]**			
	KoBoToolbox is a free and open-source suite of tools for data collection and analysis in humanitarian emergencies and other challenging environments. Most users are people working in humanitarian crises, aid professionals and researchers working in low-income countries.	Harvard Humanitarian Initiative	13 (87)
**Voozanoo [26]**			
	The Voozanoo platform is used for research (cohorts, epidemiological, or clinical studies), epidemiological surveillance, alert and response to epidemics, and prevention, screening, or coordination of care (computerized patient record and medical databases).	Epiconcept	13 (87)
**Epicollect5 [27]**			
	Epicollect5 is a mobile and web-based application for free and easy data collection. It provides modules for the generation of forms and freely hosted project websites. Data are collected (including GPS and media) using multiple devices, and all data can be viewed on a central server (via maps, tables, and charts).	Centre for Genomic Pathogen Surveillance	13 (87)
**ODK^f^ [28]**			
	ODK lets you build powerful offline forms to collect the data you need wherever it is. It supports geolocations, images, audio clips, video clips, and bar codes, as well as numerical and textual answers. ODK can evaluate complex logic to control the display prompts and impose constraints on their responses; it also supports groups of repetitive questions and data collection in multiple languages.	ODK Collect data anywhere	13 (87)
**EWARS^g^ [29,30]**			
	EWARS is designed to improve disease outbreak detection in emergency settings, such as in countries in conflict or following a natural disaster. It is a simple and cost-effective way to rapidly set up a disease surveillance system.	World Health Organization	12 (80)
**SurveyCTO [31]**			
	SurveyCTO is a reliable, secure, and scalable mobile data collection platform for researchers and professionals working in offline settings.	Dobility	12 (80)
**Afyadata [32]**			
	Afyadata is an open-source tool for collecting and submitting data from health facilities to the main server and receiving feedback from the main server. The tool provides a graphical user interface for involved health stakeholders to analyze and visualize data. It is a customized version of ODK, which has the best form management modules.	The Southern African Centre for Infectious Disease Surveillance Foundation for One Health	12 (80)
**REDCap^h^ [33]**			
	REDCap is a tool for data collectors needing to capture data offline. Data can be collected on an iPhone, iPad, Android phone, or tablet.	Vanderbilt University Medical Center	12 (80)
**Magpi [34]**			
	Magpi can be used to create forms that are responsive and look great on Android and iOS mobile and tablets in any language. It captures better data using GPS, near-field communication, signatures, bar codes, photographs, and other form responses.	Magpi	11 (73)
**Incident Tracker [35]**			
	Incident Tracker is a comprehensive way to report, track, and trend incidents. It works directly with numerous health care agencies. Incident Tracker uses the Microsoft Azure platform for the industry’s highest security and data protection levels.	McKula Inc	10 (67)
**Meningitis platform**			
	Meningitis Platform is used in Morocco for the surveillance of meningitis cases. It was developed in-country and is commonly known as meningitis platform. We identified this tool through the telephone survey, and it has no official website.	Ministry of Health (Morocco)	9 (60)
**Coconut plus (coconut surveillance) [36]**			
	Coconut Surveillance is a free and open-source software designed for malaria elimination. There are no licensing fees, and it is available at no cost. It includes an interactive SMS text messaging for case notification, a mobile software app designed to guide mobile case workers, and an analytics software app designed for surveillance and response program managers.	Research Triangle Institute International in collaboration with the President’s Malaria Initiative the Zanzibar Malaria Elimination Program.	9 (60)
**NMCSS^i^ [37]**			
	NMCSS is a tool for reporting notifiable medical conditions by all health professionals (nurses, doctors, and pathologists). The app allows for real-time reporting of infectious diseases at the point of diagnosis to local, district, provincial, and national health authorities, facilitating timely communication among all CDC personnel at various health levels.	National Institute for Communicable Diseases on behalf of the National Department of Health, South Africa	9 (60)
**Sense Followup [38]**			
	Sense Followup is a hybrid mobile app built for Android phones or tablets. It provides a simple interface that guides health workers through the processes of registering a contact and performing a follow-up.	eHealth Africa	7 (47)
**AVADAR^j^ [39,40]**			
	AVADAR is a mobile SMS text messaging–based software app designed to improve the quality and sensitivity of acute flaccid paralysis surveillance. Health care workers and key informants within hospital facilities and local communities uses AVADAR.	eHealth Africa	6 (40)
**EWORS^k^ [41]**			
	EWORS uses advanced surveillance mechanisms to detect disease outbreaks earlier than possible. EWORS allows for the electronic collection and analysis of routine clinical and nonclinical data to identify the likelihood of occurrence of a disease outbreak in a given region.	InStrat Global Health solutions	6 (40)
**mAlert**			
	The mAlert system is used for reporting notifiable diseases. It integrates health surveillance data into a single platform for analysis. We identified this tool through the telephone survey, and it has no official website.	Mozambique National Institute of Health	6 (40)
**DHIMS2^l^ [42]**			
	DHIMS2 is a customization of DHIS2 for Ghana. It is used for the management of health data at all administrative levels on Ghana.	Ghana Health Service	5 (33)
**SIS-MA (Health Information System for Monitoring and Evaluation) [43]**			
	The National Health Information System for Monitoring and Evaluation aims to support the collection, analysis, interpretation, and dissemination of health data that is used to plan public health services across Mozambique from all districts to the capital through the provinces according to the hierarchical organizational structure of the Ministry of Health.	Mozambican Open Architecture Standards and Information Systems and Jembi health systems	5 (33)
**mSERS^m^**			
	mSERS is an SMS text messaging–based tool used in Nigeria for aggregate reporting of health data. We identified this tool through the telephone survey, and it has no official website.	CDC	5 (33)
**ARGUS [44]**			
	ARGUS was designed to make the best use of limited human and financial resources for public health surveillance. It is open-source, easy to configure, and multilingual.	World Health Organization	5 (33)
**RapidSMS [45]**			
	RapidSMS is a free and open-source framework for building interactive SMS text messaging apps, which integrates tightly with Django to provide a rich reporting interface.	Innovation Team at the United Nations Children’s Fund and the RapidSMS Team	4 (27)
**mTrac [46]**			
	The mTrac system was designed for the real-time data collection, verification, accountability, and analysis of aggregate data and community engagement for the improvement of health care service delivery. mTrac is powered by RapidSMS.	Ministry of Health of Uganda	4 (27)
**FrontlineSMS [47]**			
	FrontlineSMS is a free and open-source software used by a variety of organizations to distribute and collect information via SMS text messaging. The software works without an internet connection and with a cell phone and computer.	Frontline	3 (20)

^a^Description retrieved from the official website for tools having a website.

^b^Number of attributes supported by tools based on available data.

^c^DHIS2: District Health Information Software 2.

^d^SORMAS: Surveillance Outbreak Response Management and Analysis System.

^e^eIDSR: electronic Integrated Disease Surveillance and Response.

^f^ODK: Open Data Kit.

^g^EWARS: Early Warning, Alert, and Response System.

^h^REDCap: Research Electronic Data Capture.

^i^NMCSS: Notifiable Medical Conditions Surveillance System.

^j^AVADAR: Auto-Visual Acute flaccid paralysis Detection And Reporting.

^k^EWORS: Early Warning Outbreak Response System.

^l^DHIMS2: District Health Information Management System 2.

^m^mSERS: mobile Strengthening Epidemic Response System.

### Clustering of Tools

**Figure 2:**
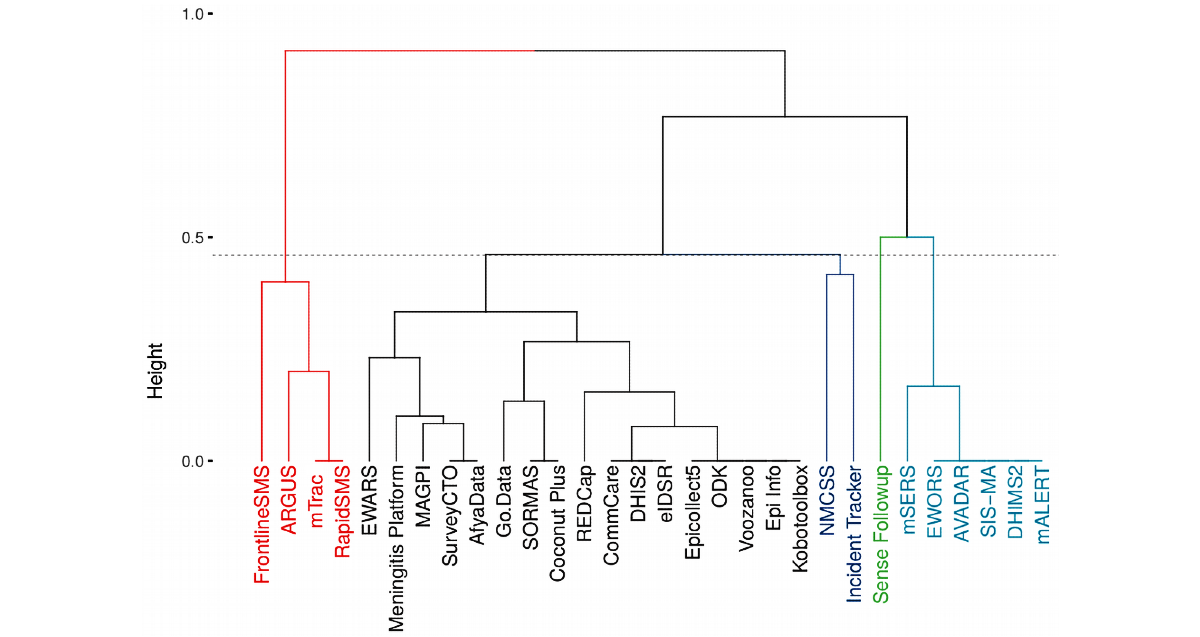
Dendrogram of 30 electronic tools for communicable disease surveillance used in 28 African countries from May 2019 to December 2020. AVADAR: Auto-Visual Acute flaccid paralysis Detection And Reporting; DHIMS2: District Health Information Management System 2; DHIS2: District Health Information Software 2; eIDSR: electronic Integrated Disease Surveillance and Response; EWARS: Early Warning, Alert, and Response System; EWORS: Early Warning Outbreak Response System; mSERS: mobile Strengthening Epidemic Response System; NMCSS: Notifiable Medical Conditions Surveillance System; ODK: Open Data Kit; REDCap: Research Electronic Data Capture; SORMAS: Surveillance Outbreak Response Management and Analysis System.

**Figure 3:**
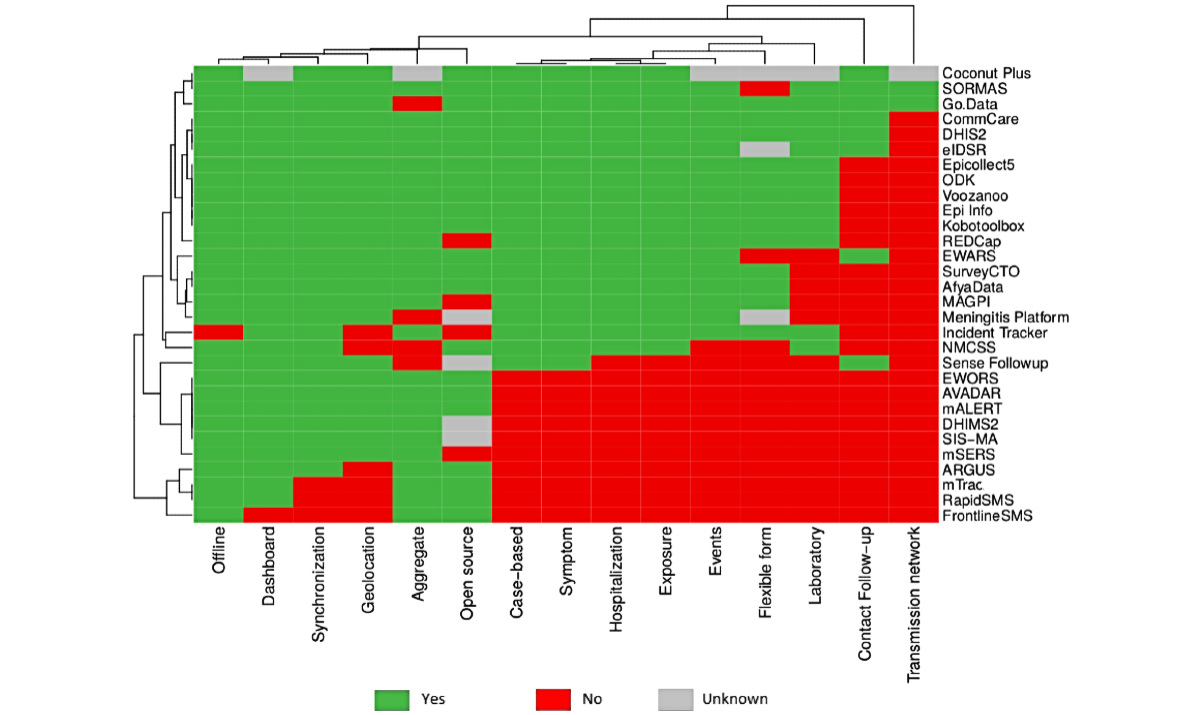
Heatmap of 30 electronic tools for communicable disease surveillance used in 28 African countries from May 2019 to December 2020. AVADAR: Auto-Visual Acute flaccid paralysis Detection And Reporting; DHIMS2: District Health Information Management System 2; DHIS2: District Health Information Software 2; eIDSR: electronic Integrated Disease Surveillance and Response; EWARS: Early Warning, Alert, and Response System; EWORS: Early Warning Outbreak Response System; mSERS: mobile Strengthening Epidemic Response System; NMCSS: Notifiable Medical Conditions Surveillance System; ODK: Open Data Kit; REDCap: Research Electronic Data Capture; SORMAS: Surveillance Outbreak Response Management and Analysis System.

We plotted the 30 electronic tools identified from 28 countries in a dendrogram. (Figure 2). Cutting the dendrogram at a height of 0.46, we grouped the tools into 5 main clusters (Figure 2). The largest cluster (in black) has 57% (17/30) of the tools that share the following attributes: case based, symptoms, hospitalization, exposures, events, aggregate, geolocation, synchronization, and dashboard. The second-largest cluster (in turquoise with 6/30, 20% of the tools) contains tools used to manage aggregate data but cannot manage case-based data. In addition, they share the following attributes: geolocation, synchronization, and dashboard. The third-largest cluster (in red with 4/30, 13% of the tools) comprises tools used to manage aggregate data but cannot manage case-based data. They also lack geolocation, synchronization, or a dashboard attribute. The smallest cluster (in green) comprises only the Sense Followup tool. It is characterized by case-based and contact follow-up attributes but lacks the following functional attributes: hospitalization, exposure, events, laboratory, and aggregate. To identify the single functional attribute that can be used to cluster the tools into 2 distinct groups, we performed a subclustering of the tools based on the 8 functional attributes only. Case-based was the most distinguishable functional attribute among the tools (Figure S1 in Multimedia Appendix 1). Figure 3 presents the clustering of the tools (horizontal dendrogram) and their attributes (vertical dendrogram) using a heat map. The 2 most distinguishable attribute clusters comprise 1 attribute only: transmission network (offered by SORMAS and Go.Data) and contact follow-up (offered by 8/30, 27% of the tools).

### Challenges to Sustain mHealth Programs

In the survey, challenges and needs to sustain mHealth programs in countries were expressed as (1) the lack of integration among the different apps used for managing health data in the country (7/27, 26% responders), (2) the need to improve the skills—comprising documenting complete and accurate data and data analysis using statistical software—of public health workers at district and regional levels through training programs within or between the different countries in Africa (6/27, 22% responders), (3) the lack of stable internet connectivity to synchronize data between mobile devices and the server (2/27, 7% responders), and (4) the lack of sustainability for continuous use of a tool after its initial piloting phase (2/27, 7% responders).

## Discussion

### Principal Results

We identified 30 digital tools for surveillance or outbreak management of communicable diseases used in Africa and a comprehensive set of 15 attributes. However, none of the 30 tools included all 15 attributes. The tools that supported 14 of the attributes were SORMAS, Go.Data, CommCare, and DHIS2. Unlike CommCare, which is a generic multipurpose tool for collecting data in a variety of fields such as research, agriculture, and international development, the other tools were developed mainly for public health use. This may explain why they supported most of the relevant attributes. On the basis of our finding from clustering, the single functional attribute that distinguished the tools into 2 main groups is the possibility for case-based reporting. Case-based surveillance has epidemiological benefits over aggregate surveillance, such as facilitating the assessment of risk factors, routes of transmission, and data quality; it also allows for immediate reporting upstream, continuous updating, and addition and correction of information, whereas for aggregate reporting, this is not possible or only possible to a very limited extent. Some of the tools supported disease-specific attributes that were not among the list of the 15 attributes evaluated. For example, DHIS2 has a module for the supply chain management of medicine. SORMAS integrates disease-specific surveillance and case management features. These include disease control measures and the management of prescriptions, treatments, and clinical courses. AfyaData, on the other hand, offers functionalities for both human and animal surveillance.

Experts considered 2 important functions as essential for responding to outbreaks: contact follow-up and visualization of chains of transmission. These functions were properly represented in 2 tools only, SORMAS and Go.Data. When isolation is enforced, contact follow-up can reduce the number of secondary cases caused by each case [48]. As contact data are usually large and complex, functionalities to visualize, filter, and compute indicators from the transmission network data may help public health officials identify superspreading events of cross-border transmission chains, thus prioritizing intervention measures. The attributes present in most tools are the ability to function offline and a dashboard for epidemiological indicators. The study participants perceived the ability of tools to work offline and synchronize with the server whenever internet connectivity is available as an important feature of the tools. This may reflect the fact that digital communication infrastructure in public health services in many areas of Africa is not reliable and continuous enough to rely solely on continuous web-based services. Furthermore, a real-time dashboard is an efficient feature to assist response coordination teams in deciding and monitoring intervention measures, especially in an outbreak situation where a delay of just 1 day can significantly reduce the effect of certain control measures.

### Limitations

Although we received responses only from 52% (28/54) of African countries for our survey, our literature review was not limited to those. Therefore, we believe that it is unlikely to have systematically missed any kind of tools, which would have resulted in a substantially different conclusion. For some tools, neither the interviewee nor any other available source of information could confirm the absence or presence of a specific attribute. As this was only the case for <3% (12/450) of tool-attribute combinations, the impact on the overall findings appears small. For tools that we identified and mapped with the information obtained from literature search only (ARGUS, Voozanoo, Epicollect5, Incident Tracker, and REDCap [Research Electronic Data Capture]), we were unable to validate whether they were in actual use in any African country. It should also be mentioned that the field of mHealth has been undergoing accelerated change since 2015; thus, it is to be expected that the findings of this study may change in the medium to long term.

### Systematic Assessments of Digital Tools for COVID-19 Response

Since the beginning of the COVID-19 outbreak, researchers and organizations have conducted systematic assessments of digital tools that can be used for COVID-19 outbreak response [49-51]. Some of the complementary features covered by these assessments, in addition to the general surveillance attributes assessed in this review, are remote monitoring of symptoms whereby patients or contacts can self-notify their symptoms during the follow-up period [52,53] and electronic immunization registries to plan and manage COVID-19 vaccine delivery and immunization programs [54]. The increased availability of these features in response to COVID-19 may indicate the demand for these attributes to be covered by or integrated into digital surveillance for other communicable or vaccine-preventable diseases.

### Recommendations for Future Research

Future research on mHealth could investigate other attributes necessary for mHealth programs that we did not consider in this study, such as the 2 features identified in the assessments of digital tools for COVID-19 response, financial cost for implementation and maintenance of a tool, data protection standards, data security audits, and multiple indicators of the global goods maturity matrix developed by Digital Square [55,56]. Factors associated with the challenges in sustaining mHealth programs identified in this study, such as data quality, sustainability, and integration, could be investigated in future studies.

### Conclusions

However, at the moment, our findings can support public health institutions in choosing the most appropriate existing tools that suit their needs, or assist developers in including relevant attributes into future tools, by highlighting some key attributes to be considered. From the challenges identified by both the systematic review and the survey, we would like to emphasize the following measures to sustain mHealth programs: improving internet connectivity for mobile devices, improving integration between tools or apps to facilitate data sharing, consistent supervision of users in the field to ensure data quality, and measures (such as maintenance, user support, and funding) to ensure sustainable use of tools or apps after the initial piloting phase. Among the large number of tools identified, only a few offer a comprehensive set of attributes as identified during our review and survey. This challenges users by being restricted to a limited set of functions per tool and having to use multiple tools in parallel to cover a larger scope of functional and nonfunctional attributes. Only 4 tools (SORMAS, Go.Data, CommCare, and DHIS2) cover a sufficiently complete set of attributes to offer an integrated and comprehensive digital support for epidemic control as in the current COVID-19 pandemic. To have a digital solution covering all the attributes evaluated in this study, any of the 4 aforementioned tools could be further developed with minimal resources compared with the others.

## Appendix

Multimedia Appendix 1 Names of countries that responded to the telephone survey, dendrogram of the tools clustered based on 8 functional attributes, flow chart of identified relevant attributes, and description of attributes excluded from the study.

## Abbreviations

DHIS2: District Health Information Software 2
IDSR: Integrated Disease Surveillance and Response
mHealth: mobile health
SORMAS: Surveillance Outbreak Response Management and Analysis System
WHO: World Health Organization
