# Supplementary material for: Availability and Suitability of Digital Health Tools in Africa for Pandemic Control: Scoping Review and Cluster Analysis
Source: JMIR Public Health Surveill. 2021 Dec 23;7(12):e30106. doi: 10.2196/30106 (PMC8738990; doi:10.2196/30106)
Supplement: Multimedia Appendix 1 [file publichealth_v7i12e30106_app1.pdf]

Table S1. Twenty eight countries that responded to the telephone survey and the corresponding number of participants. We conducted the survey from May 2019 to December 2020.

Country	Number of participants
Benin	2
Burkina Faso	1
Burundi	1
Cameroon	1
The Democratic Republic of the Congo	1
Equatorial Guinea	1
Ethiopia	1
Gabon	1
Ghana	2
Ivory Coast	1
Liberia	2
Malawi	2
Mauritania	2
Mauritius	1
Morocco	2
Mozambique	2
Namibia	1
Niger	1
Nigeria	3
Republic of the Congo	1
Rwanda	1
São Tomé and Príncipe	1
South Africa	1
Sudan	2
Tanzania	3
Uganda	3
Zambia	2
Zimbabwe	3

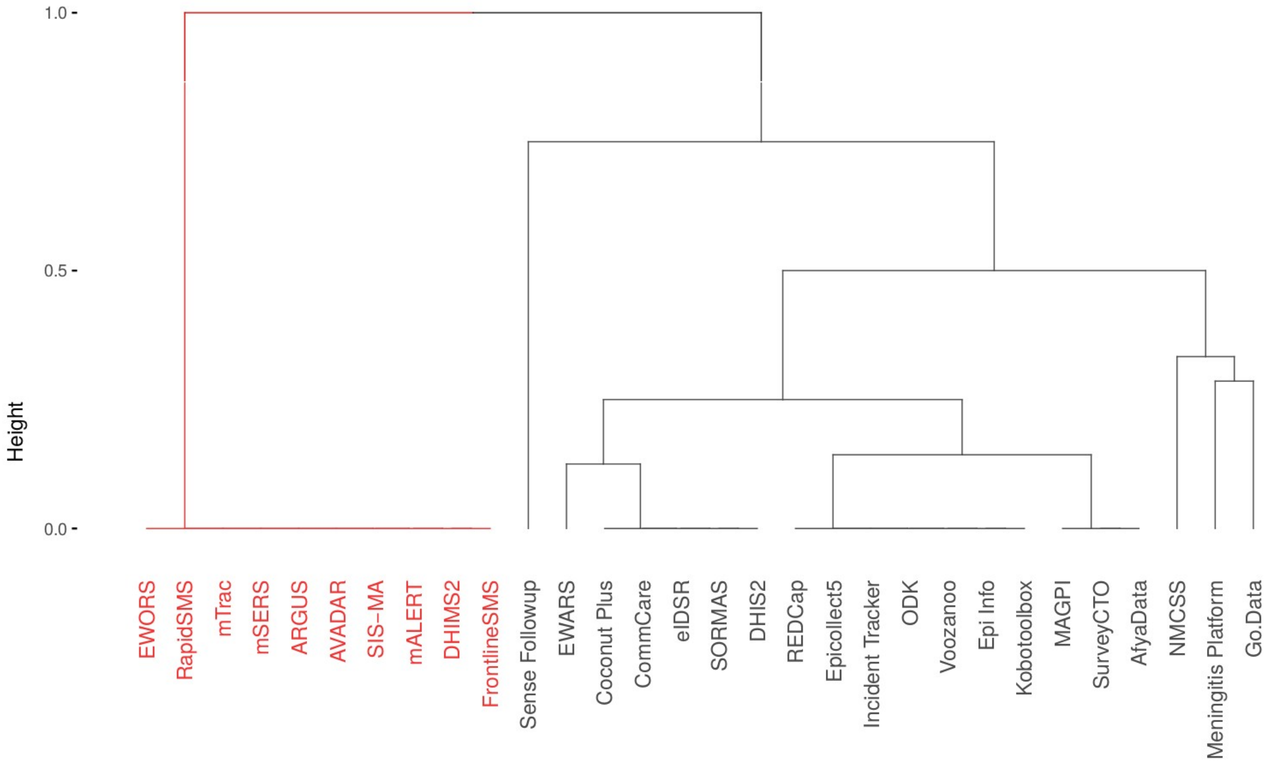

Figure S1. Dendrogram of 30 electronic tools for communicable diseases surveillance used in 28 Africa countries from January 2010 to December 2020. We used hierarchical clustering with Jaccard similarity coefficient to cluster the tools based on eight functional attributes. The black cluster corresponds to case-based tools while the red corresponds to aggregate tools. The values on the vertical axis (height) are the distances or dissimilarities between combined tools while the leaves are the names of the tools.

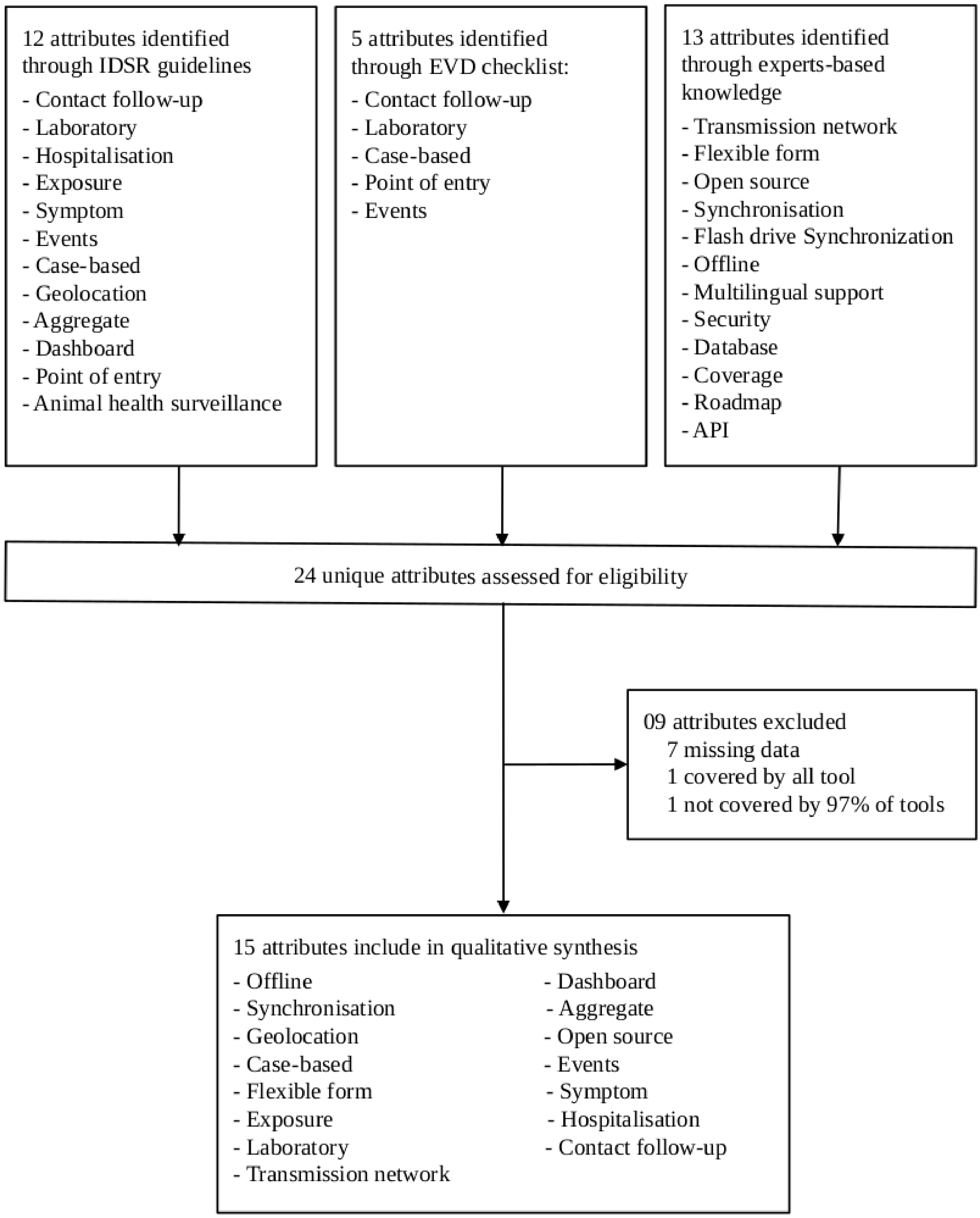

Figure S2. Flow chart to identify relevant attributes of electronic tools for communicable diseases surveillance with publications or used in 28 African countries from January 2010 to December 2020.

Table S2. Description of 9 eligible attributes of electronic tools for communicable diseases excluded from the study, January 2010 to December 2020.

Attribute label	Attribute description
Flash drive Synchronization	Data can be transferred from application on mobile device or PC to a flash drive and later synchronize the data with server
Multilingual support	Tool can be used in more than one user profile languages
Security	The type of encryption used for data access and transfer
Database	The database name
Coverage	The number of countries, regions, and districts using the tool
Roadmap	Tool has a roadmap to provide an overview of the development process
API	Tool has an Application Programming Interface (API) to exchange its data with another application
Point of entry (POE)	Tool can be used for surveillance at boarders or ports of entry
Animal health surveillance	Tool can be used for surveillance of human and animal disease
